# Exponential Time Differencing Algorithm for Pulse-Coupled Hodgkin-Huxley Neural Networks

**DOI:** 10.3389/fncom.2020.00040

**Published:** 2020-05-08

**Authors:** Zhong-qi Kyle Tian, Douglas Zhou

**Affiliations:** School of Mathematical Sciences, MOE-LSC, Institute of Natural Sciences, Shanghai Jiao Tong University, Shanghai, China

**Keywords:** Hodgkin-Huxley, exponential time differencing method, efficiency, pulse-coupled, second-order

## Abstract

The exponential time differencing (ETD) method allows using a large time step to efficiently evolve stiff systems such as Hodgkin-Huxley (HH) neural networks. For pulse-coupled HH networks, the synaptic spike times cannot be predetermined and are convoluted with neuron's trajectory itself. This presents a challenging issue for the design of an efficient numerical simulation algorithm. The stiffness in the HH equations are quite different, for example, between the spike and non-spike regions. Here, we design a second-order adaptive exponential time differencing algorithm (AETD2) for the numerical evolution of HH neural networks. Compared with the regular second-order Runge-Kutta method (RK2), our AETD2 method can use time steps one order of magnitude larger and improve computational efficiency more than ten times while excellently capturing accurate traces of membrane potentials of HH neurons. This high accuracy and efficiency can be robustly obtained and do not depend on the dynamical regimes, connectivity structure or the network size.

## 1. Introduction

The Hodgkin-Huxley (HH) model (Hodgkin and Huxley, 1952; Hassard, 1978; Dayan and Abbott, 2003) is a classical neuron model, originally proposed to describe the behaviors of action potentials of the squid's giant axon. It provides a useful mechanism that accounts for the detailed generation of action potentials and the existence of the absolute refractory periods. It also serves as the foundation for other neuron models such as the one that can describe the behaviors of bursting and adaption (Pospischil et al., 2008). However, the HH equations are so complicated that it is difficult to study its properties analytically such as the Hopf bifurcation and chaotic dynamics (Aihara, 1986; Hansel and Sompolinsky, 1996; Guckenheimer and Oliva, 2002; Lin, 2006). Therefore, its investigation often relies on numerical simulations, for example, by the Runge-Kutta (RK) methods.

There are several difficulties to design an efficient and accurate numerical algorithm for the HH neural network, especially when the network size is large. First, when an HH neuron driven by external input generates an action potential (the interval of action potential is called spike period in this work), the HH neuron equations become stiff. Regular RK methods have to use very small time step to satisfy the requirement of numerical stability (Guckenheimer and Oliva, 2002; Börgers et al., 2005; Kassam and Trefethen, 2005; Börgers and Nectow, 2013). This small time step will significantly increase the computational cost when studying long time behavior of large-scale HH networks such as chaotic attractor dynamics or collecting reliable statistical information of HH neurons such as the distribution of inter-spike-intervals.

For more realistic situations, the neurons are generally driven by stochastic spike input and the interaction term is usually modeled by a Dirac delta function (pulse-coupled), while the spike-induced conductance dynamics are modeled by an *alpha* function (Somers et al., 1995; Hansel et al., 1998; Sun et al., 2009). These make the system become non-smooth and event-driven, while providing challenges for the design of efficient numerical simulation algorithms. For instance, it is impossible to predetermine the synaptic spike times since they are convoluted with neurons' trajectories themselves. As a result, one has to evolve the HH network by ignoring the spike interactions among neurons and then use spike-spike interaction to amend the neurons' trajectories at the end of the time step (Hansel et al., 1998; Brette et al., 2007). Without a careful recalibration for the neuronal spikes, the numerical algorithm often suffers from the issue of instability or relatively low numerical accuracy.

The exponential time differencing (ETD) method (Hochbruck et al., 1998; Cox and Matthews, 2002; Kassam and Trefethen, 2005; de la Hoz and Vadillo, 2008; Nie et al., 2008; Hochbruck and Ostermann, 2010) is proposed for efficient simulation of stiff ordinary differential equations (ODEs). The basic idea is to decompose the ODEs into a linear stiff part and a nonlinear non-stiff part. Then, the linear stiff part can be solved by using the integrating factor method, while the nonlinear non-stiff part can be approximated by numerical quadrature (Cox and Matthews, 2002). A second-order ETD (ETD2) method for HH neural networks has been proposed in a recent work (Börgers and Nectow, 2013), which allows using a large time step to raise computational efficiency. In Börgers and Nectow (2013), the HH equations are linearly approximated in each time step, and then solved analytically over the time step. The ETD2 method proposed in Börgers and Nectow (2013) is a reduced situation of that in Cox and Matthews (2002), but it is difficult to generalize to higher-order cases, e.g., the fourth-order ETD method. Besides, although the ETD2 method proposed in Börgers and Nectow (2013) is proven to be unconditionally stable for HH system, it will be inaccurate using a large time step (Börgers and Nectow, 2013).

In this work, we first provide an ETD2 method following the idea proposed in Cox and Matthews (2002) to evolve a pulse-coupled HH neural network driven by stochastic spike input. Note that the stiffness of HH equations are quite different, especially between the spike and non-spike periods, and we find that the ETD2 method may introduce a relatively large error in the membrane potentials in the non-stiff period if using the same time step as that in the stiff period. We then design an adaptive ETD2 method (AETD2) that using different decompositions of the linear and non-linear parts in stiff and non-stiff periods. In addition, for the situation where neurons generate spikes in the time step, the effects of the spikes are carefully recalibrated in our AETD2 method to achieve a second-order numerical accuracy. Our AETD2 method is capable of using a large time step, while achieving the same high accurate traces of membrane potential of each neuron as the second-order RK (RK2) method using a very small time step. It can improve computational efficiency more than one order of magnitude compared with the RK2 method. This high numerical accuracy and computational efficiency can be achieved over a wide range of dynamical regimes and does not depend on the network connectivity or size.

## 2. Materials and Methods

### 2.1. The Model

The dynamics of the *i*th neuron of an HH neural network is governed by

(1) $$\begin{array}{rc} C\frac{dV_{i}}{dt} & =-\left(V_{i}-V_{Na}\right)G_{Na}m_{i}^{3}h_{i}-\left(V_{i}-V_{K}\right)G_{K}n_{i}^{4}\\ \;-\left(V_{i}-V_{L}\right)G_{L}+I_{i}^{\text{input}}, \end{array}$$

(2) $$\begin{array}{rc} \frac{dz_{i}}{dt} & =\left(1-z_{i}\right)\alpha_{z}\left(V_{i}\right)-z_{i}\beta_{z}\left(V_{i}\right),\text{for}z=m,h,n, \end{array}$$

where *C* is the cell membrane capacitance, *V*_*i*_ is the membrane potential, *m*_*i*_, *h*_*i*_, and *n*_*i*_ are gating variables for sodium and potassium currents, respectively (Dayan and Abbott, 2001). The parameters *V*_*Na*_, *V*_*K*_, and *V*_*L*_ are the reversal potentials for the sodium, potassium, and leak currents, respectively, *G*_*Na*_, *G*_*K*_, and *G*_*L*_ are the corresponding maximum conductances. The form of α_*z*_ and β_*z*_ are set as (Dayan and Abbott, 2001): α_*m*_(*V*) = (0.1*V*+4)/(1−exp(−0.1*V*−4)), β_*m*_(*V*) = 4exp(−(*V*+65)/18), α_*h*_(*V*) = 0.07exp(−(*V*+65)/20), β_*h*_(*V*) = 1/(1+exp(−3.5−0.1*V*)), α_*n*_(*V*) = (0.01*V*+0.55)/(1−exp(−0.1*V*−5.5)), and β_*n*_(*V*) = 0.125exp(−(*V*+65)/80).

The input current $I_{i}^{\text{input}}$ is given by

(3) $$\begin{array}{r} I_{i}^{\text{input}}=-G_{i}^{E}\left(t\right)\left(V_{i}-V_{G}^{E}\right)-G_{i}^{I}\left(t\right)\left(V_{i}-V_{G}^{I}\right), \end{array}$$

where $G_{i}^{E}$ and $G_{i}^{I}$ are excitatory and inhibitory conductances, respectively, $V_{G}^{E}$ and $V_{G}^{I}$ are the corresponding reversal potentials. The dynamics of conductance $G_{i}^{Q}$, *Q* = *E, I*, is governed by

(4) $$\begin{array}{rc} \frac{dG_{i}^{Q}}{dt} & =-\frac{G_{i}^{Q}}{\sigma_{r}^{Q}}+H_{i}^{Q}, \end{array}$$

(5) $$\begin{array}{rc} \frac{dH_{i}^{Q}}{dt} & =-\frac{H_{i}^{Q}}{\sigma_{d}^{Q}}+F^{Q}\underset{l}{\sum }\delta \left(t-s_{il}\right)+\underset{j}{\sum }S_{ij}^{Q}\underset{l}{\sum }\delta \left(t-\tau_{jl}\right), \end{array}$$

where $H_{i}^{Q}$ is an auxiliary dynamical variable to make the conductance $G_{i}^{Q}$ as a continuous function, δ(·) is the Dirac delta function, *s*_*il*_ is the spike time of the feedforward Poisson input with strength *F*^*Q*^ and rate ν, τ_*jl*_ is the *l*th spike time of the *j*th neuron, and $\sigma_{d}^{Q}$ and $\sigma_{r}^{Q}$ are slow decay and fast rise time scale, respectively. Each neuron is either excitatory or inhibitory and its coupling strength is labeled by its type *E* or *I*, respectively. For example, $S_{ij}^{E}$ ($S_{ij}^{I}$) is the coupling strength from the *j*th excitatory (inhibitory) neuron to its postsynaptic *i*th neuron. By analytically solving Equations (4) and (5), the spike-induced conductance change can be explicitly expressed as

(6) $$\begin{array}{r} G\left(\sigma_{d}^{Q},\sigma_{r}^{Q},t\right)=\frac{\sigma_{d}^{Q}\sigma_{r}^{Q}}{\sigma_{d}^{Q}-\sigma_{r}^{Q}}\left(e^{-t/\sigma_{d}^{Q}}-e^{-t/\sigma_{r}^{Q}}\right)\Theta \left(t\right), \end{array}$$

where Θ(·) is the Heaviside function. For all neurons, we take *F*^*E*^ = *f* and *F*^*I*^ = 0. The model parameters are *C* = 1μF·cm^−2^, *V*_*Na*_ = 50 mV, *V*_*K*_ = −77 mV, *V*_*L*_ = −54.387 mV, $G_{Na}=120\text{mS}\cdot {\text{cm}}^{-2}$, $G_{K}=36\text{mS}\cdot {\text{cm}}^{-2}$, $G_{L}=0.3\text{mS}\cdot {\text{cm}}^{-2}$, $V_{G}^{E}=0$ mV, $V_{G}^{I}=-80$ mV, $\sigma_{r}^{E}=0.5$ ms, $\sigma_{d}^{E}=3.0$ ms, $\sigma_{r}^{I}=0.5$ ms, and $\sigma_{d}^{I}=7.0$ ms (Dayan and Abbott, 2001).

The voltage *V*_*i*_ evolves continuously according to Equations (1) and (2). When it reaches the firing threshold *V*^th^ = −50 mV (Sun et al., 2009), we say the *i*th neuron generates a spike at this time, say τ_*il*_. Then it will trigger its postsynaptic *j*th neuron's conductance change in the form of $S_{ji}^{Q}G\left(\sigma_{d}^{Q},\sigma_{r}^{Q},t-\tau_{il}\right)$, *Q* = *E, I*. For the ease of discussion about our algorithm design, we consider an all-to-all connected network with $S_{ij}^{Q}=S/N$, where *Q* = *E, I*, *S* is the coupling strength, and *N* is the total number of neurons in the network. Note that our algorithm can be easily extended to networks with more complicated connectivity structure.

### 2.2. Runge-Kutta Method

Without loss of generality, we consider the RK2 method as the benchmark and compare it with the ETD methods. We first introduce the RK2 method to evolve the HH neural network with a fixed time step Δ*t*, for example, to evolve the system from time *t* = *t*_*k*_ = *kΔt* to *t* = *t*_*k*+1_ = (*k*+1)Δ*t*. Since the synaptic spike times in [*t*_*k*_, *t*_*k*+1_] can not be predetermined, one has to evolve the network without considering synaptic spike interactions and reconsider their effects by using spike-spike interactions at the end of time step (Hansel et al., 1998; Brette et al., 2007).

Due to the pulse-coupled dynamics in Equation (5), the numerical accuracy may be very low if the spike timing is not well estimated. For example, suppose that a presynaptic spike fired at $\tilde{t}$ between *t*_*k*_ and *t*_*k*+1_. If one simply assigns it to be the end of time step *t*_*k*+1_, then the error of the spike-induced conductance change is

(7) $$\begin{array}{rcl} \frac{S}{N}\left[G\left(\sigma_{d}^{Q},\sigma_{r}^{Q},t-\tilde{t}\right)-G\left(\sigma_{d}^{Q},\sigma_{r}^{Q},t-t_{k+1}\right)\right] & = & O\left(t_{k+1}-\tilde{t}\right)\\ \;= & O\left(\Delta t\right),Q=E,I. \end{array}$$

Therefore, the error with the magnitude of Δ*t* will be introduced when the system evolves to *t* = *t*_*k*+1_.

We now solve the above issue arising from the pulse-coupled dynamics to achieve a second-order numerical accuracy. First, we evolve the HH neural network without considering the feedforward and synaptic spikes during the time interval [*t*_*k*_, *t*_*k*+1_]. Then, at time *t* = *t*_*k*+1_, some neuron's voltage may be above the threshold, i.e., generating a spike, say neuron *i*, if *V*_*i, k*_<*Vth* and *V*_*i, k*+1_≥*Vth* where *V*_*i, k*_ and *V*_*i, k*+1_ represent *V*_*i*_(*t*_*k*_) and *V*_*i*_(*t*_*k*+1_), respectively. The spike time, say τ_*il*_, can be estimated following the idea proposed in Hansel et al. (1998) and Shelley and Tao (2001). The neuron's membrane potential during the time interval can be approximated by a linear interpolation:

(8) $$\begin{array}{r} V_{i}\left(t\right)\approx V_{i,k}+\frac{V_{i,k+1}-V_{i,k}}{\Delta t}\left(t-t_{k}\right), \end{array}$$

and the spike time τ_*il*_ can be estimated by solving the equation:

(9) $$\begin{array}{r} V^{\text{th}}=V_{i,k}+\frac{V_{i,k+1}-V_{i,k}}{\Delta t}\left(\tau_{il}-t_{k}\right). \end{array}$$

Since there may be some neurons firing and some feedforward spikes emitting during the time interval and they will induce the conductance change, the conductance should be then recalibrated. When the neuron firing and the feedforward spikes are not considered, the conductance variables in such cases, denoted by ${\tilde{G}}^{Q}$ and ${\tilde{H}}^{Q}$, *Q* = *E, I*, are

(10) $$\begin{array}{rc} {\tilde{H}}_{j,k+1}^{Q} & =H_{j,k}^{Q}e^{-\Delta t/\sigma_{d}^{Q}}, \end{array}$$

(11) $$\begin{array}{rc} {\tilde{G}}_{j,k+1}^{Q} & =G_{j,k}^{Q}e^{-\Delta t/\sigma_{r}^{Q}}+H_{i,k}^{Q}G\left(\sigma_{d}^{Q},\sigma_{r}^{Q},\Delta t\right), \end{array}$$

and the conductance variables are then recalibrated by taking into account the neuron firing and the feedforward spikes as

(12) $$\begin{array}{rc} H_{j,k+1}^{Q} & ={\tilde{H}}_{j,k+1}^{Q}+F^{Q}\underset{t_{k}<s_{jl}\leq t_{k+1}}{\sum }e^{-\left(t_{k+1}-s_{jl}\right)/\sigma_{d}^{Q}}\\ \;+\frac{S}{N}\underset{i}{\sum }\underset{t_{k}<\tau_{il}\leq t_{k+1}}{\sum }e^{-\left(t_{k+1}-\tau_{il}\right)/\sigma_{d}^{Q}}, \end{array}$$

(13) $$\begin{array}{rc} G_{j,k+1}^{Q} & ={\tilde{G}}_{j,k+1}^{Q}+F^{Q}\underset{t_{k}<s_{jl}\leq t_{k+1}}{\sum }G\left(\sigma_{d}^{Q},\sigma_{r}^{Q},t_{k+1}-s_{jl}\right)\\ \;+\frac{S}{N}\underset{i}{\sum }\underset{t_{k}<\tau_{il}\leq t_{k+1}}{\sum }G\left(\sigma_{d}^{Q},\sigma_{r}^{Q},t_{k+1}-\tau_{il}\right), \end{array}$$

for *j* = 1, 2, …, *N*. A detailed algorithm of the RK2 method is given in Algorithm 1.

**Algorithm 1:** RK2 algorithm

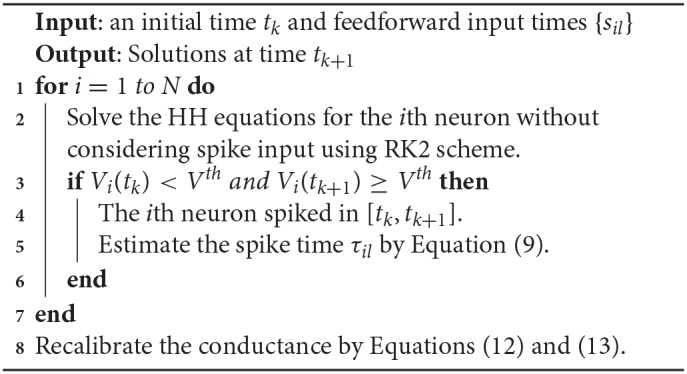

We show that the above algorithm can indeed achieve a second-order numerical accuracy as follows. If there are no feedforward or synaptic spikes, then all the dependent variables are infinitely differentiable and the RK2 method can achieve an error of order *O*(Δ*t*^2^). For the time step that contains feedforward or synaptic spikes, an error of order *O*(Δ*t*) is introduced in the conductance with the form of $G^{Q}-{\tilde{G}}^{Q},Q=E,I$. Nevertheless, the dependent variables of *V, m, h*, and *n* can have an error of order *O*(Δ*t*^2^). The synaptic spike times are estimated by a linear interpolation and also have an error of order *O*(Δ*t*^2^). After recalibration shown in Equations (12) and (13), the conductance variables *G*^*Q*^ and *H*^*Q*^, *Q* = *E, I* can achieve numerical accuracy of second-order at the end of the time step. Therefore, all the dependent variables *V, m, h, n, G*^*E*^, *G*^*I*^, *H*^*E*^, and *H*^*I*^ that are numerically solved in each time step have an error of order *O*(Δ*t*^2^) (see below for verification of numerical results).

### 2.3. Exponential Time Differencing Method

Exponential time differencing method is proposed to solve the stiff problem in differential equations by decomposing the system into a linear stiff term and a nonlinear non-stiff term (Hochbruck et al., 1998; Cox and Matthews, 2002; Kassam and Trefethen, 2005; Nie et al., 2008). Following this idea, we propose the ETD schemes for HH Equations (1) and (2) below. As illustrated in Algorithm 1, each neuron in the HH network is evolved independently and their conductances are recalibrated at the end of time step. Thus, one can first derive an ETD scheme for a single HH neuron and then consider the spike interactions among neurons, and obtain an ETD scheme for the numerical evolution of an HH neural network.

Consider the evolution of a single HH neuron from *t*_*k*_ to *t*_*k*+1_. The first step of the ETD method is to rewrite Equations (1) and (2) as

(14) $$\begin{array}{r} \frac{dz}{dt}=c_{z}z+F_{z},\text{for}z=V,m,h,n, \end{array}$$

where

(15) $$\begin{array}{r} c_{V}=\left(-G_{Na}m_{k}^{3}h_{k}-G_{K}n_{k}^{4}-G_{L}\right)/C, \end{array}$$

(16) $$\begin{array}{r} c_{z}=-\alpha_{z}\left(V_{k}\right)-\beta_{z}\left(V_{k}\right),\text{for}z=m,h,n, \end{array}$$

(17) $$\begin{array}{rc} F_{V}\left(t,V,m,h,n\right) & =[-(V-V_{Na})G_{Na}m^{3}h-(V-V_{K})G_{K}n^{4}\\ \;-(V-V_{L})G_{L}+I^{\text{input}}]/C-c_{V}V \end{array}$$

and

(18) $$\begin{array}{r} F_{z}\left(t,V,m,h,n\right)=\left(1-z\right)\alpha_{z}\left(V\right)-z\beta_{z}\left(V\right)-c_{z}z,\text{for}z=m,h,n, \end{array}$$

where *z*_*k*_ represents *z*(*t*_*k*_) for *z* = *V, m, h, n* of this neuron. Here, *F*_*z*_(*t, V, m, h, n*) is actually a function of *t, V*, and *z* for *z* = *m, h, n*, but we write in this way for ease of illustration. Note that the linear coefficient *c*_*z*_ in Equation (14) is a constant value in the *k*th time step [*t*_*k*_, *t*_*k*+1_] and is updated with respect to *k*. Multiplying Equation (14) by an integrating factor $e^{-c_{z}t}$ and taking integral from *t*_*k*_ to *t*_*k*+1_, we obtain

(19) $$\begin{array}{rcl} z_{k+1} & = & z_{k}e^{c_{z}\Delta t}+e^{c_{z}\Delta t}\overset{\Delta t}{\underset{0}{\int }}e^{-c_{z}\tau }F_{z}(t_{k}\\ \;+\tau ,V(t_{k}+\tau ),m(t_{k}+\tau ),h(t_{k}+\tau ),n(t_{k}+\tau ))d\tau \end{array}$$

for *z* = *V, m, h*, and *n*.

The next step of the ETD method is to derive proper approximations to the above integration. We take a second-order ETD formula with RK time stepping which was proposed as ETD2RK method in Cox and Matthews (2002). Let,

(20) $$\begin{array}{r} a_{z,k}=z_{k}e^{c_{z}\Delta t}+F_{z,k}\left(e^{c_{z}\Delta t}-1\right)/c_{z}, \end{array}$$

and approximate *F*_*z*_ during the time interval [*t*_*k*_, *t*_*k*+1_] by

(21) $$\begin{array}{r} F_{z}(t_{k}+\tau ,V\left(t_{k}+\tau \right),m\left(t_{k}+\tau \right),h\left(t_{k}+\tau \right),n\left(t_{k}+\tau \right)\\ =F_{z,k}+\tau \left(F_{z}\left(t_{k+1},a_{V,k},a_{m,k},a_{h,k},a_{n,k}\right)-F_{z,k}\right)/\Delta t+O\left(\Delta t^{2}\right), \end{array}$$

for *z* = *V, m, h*, and *n*, where *F*_*z, k*_ represents *F*_*z*_(*t*_*k*_, *V*_*k*_, *m*_*k*_, *h*_*k*_, *n*_*k*_). Substituting the above approximation into Equation (19) yields the ETD2 scheme (the ETD method which has second-order numerical accuracy) which is given by

(22) $$\begin{array}{rcl} z_{k+1} & = & a_{z,k}+[F_{z}\left(t_{k+1},a_{V,k},a_{m,k},a_{h,k},a_{n,k}\right)\\ \;- & F_{z,k}]\left(e^{c_{z}\Delta t}-1-c_{z}\Delta t\right)/c_{z}^{2}\Delta t, \end{array}$$

for *z* = *V, m, h*, and *n*. The procedure of the ETD2 algorithm for an HH neural network is similar to that of the RK2 algorithm given in Algorithm 1, but the RK2 scheme is replaced by the ETD2 scheme in Equation (22).

### 2.4. Adaptive Exponential Time Differencing Method

The ETD2 method can indeed use a large time step to improve computational efficiency, but we find that it will introduce relatively large error in the trajectories of neurons' membrane potentials and even lead to the missing of action potentials (see below for numerical results). In addition, the number of the missed action potentials in the ETD2 method can grow with the increase of time steps compared with the RK2 method using a small time step. Thus, it is important to design an efficient but also reliable ETD method to solve this issue.

**Figure 1:**
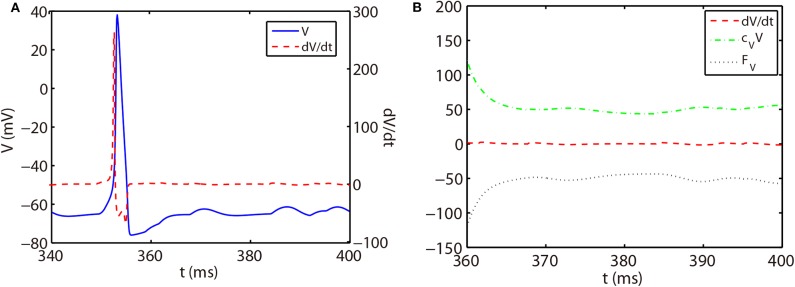
**(A)** Trajectory of voltage (blue solid curve) and the slope of voltage (red dashed curve) for a single HH neuron. **(B)** Trajectory of the slope of voltage (red dashed curve), linear part *c*_*V*_*V* (green dash-dotted curve), and nonlinear part *F*_*V*_ (black dotted curve) in Equation (14) for the non-spike period. The time interval of **(B)** zooms into the later part of **(A)**.

As shown in Figure 1A, the slope of voltage has a very large value when the neuron generates an action potential (spike period) and quickly reduces to a value around zero in the non-spike period until the next spike time. Therefore, the stiffness of HH equations is quite different between spike and non-spike periods. In the non-spike period, the slope of voltage is almost zero, while the linear and nonlinear parts in Equation (14) have a much larger absolute value and nearly cancel each other out as shown in Figure 1B. Therefore, the decomposition in Equation (14) may not be appropriate in the non-spike period since both the linear and nonlinear parts become stiff while the summation of them is indeed non-stiff. Based on this, we propose a different decomposition in the non-stiff period from that in the stiff period: taking *c*_*z*_ = 0 and the whole right hand side of Equation (14) as the nonlinear part. For such a decomposition, the ETD2 scheme reduces to the RK2 scheme in the non-stiff period.

**Figure 2:**
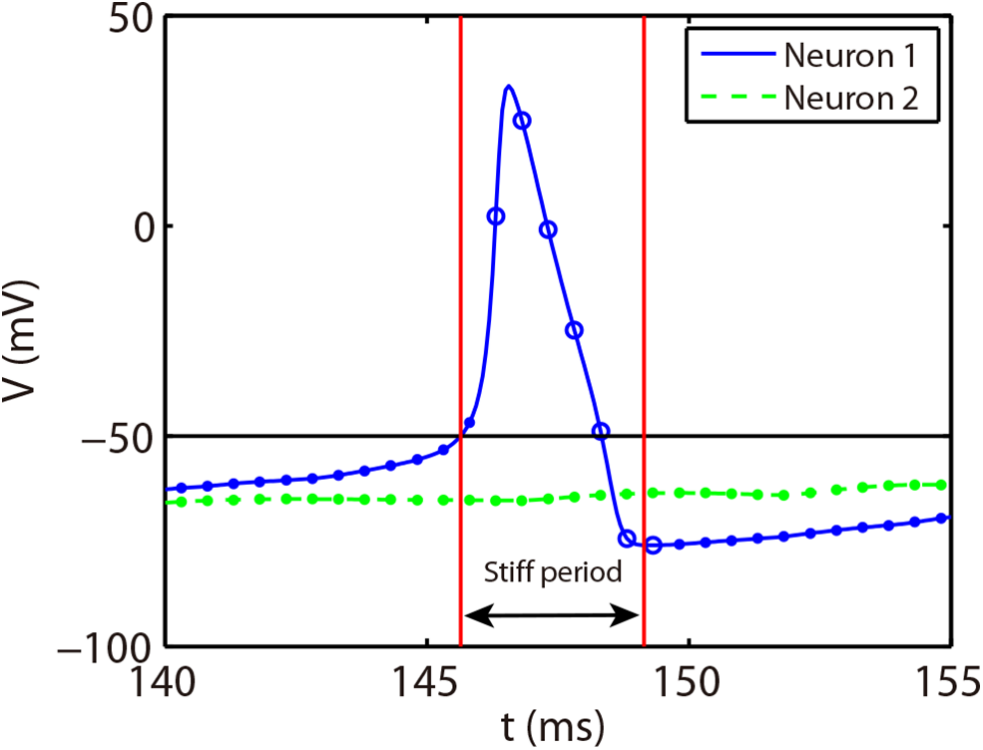
Illustration of the AETD2 method. After neuron 1 fires a spike, we use the ETD2 scheme to evolve the HH equations for neuron 1 during the stiff period indicated by the red vertical lines, while the HH equations for neuron 2 is evolved using RK2 scheme since neuron 2 is in the non-stiff period. The starting point of the stiff period is determined as the spike time and it lasts for the following about 3.5 ms. The circles and dots indicate the time nodes where we use the ETD2 and RK2 schemes, respectively.

The stiff period of HH equations can be clearly identified as shown in Figure 1A and is defined as follows. For each spike event, the starting point of the stiff period is determined as the spike time when the voltage reaches the firing threshold *Vth* = −50 mV and the interval of stiff period is chosen as 3.5 ms which is sufficient long to cover the highly stiff region of the spike. Based on the above observation, we give our AETD2 method for HH neural network as following: each neuron is evolved using ETD2 scheme if it is in the stiff period and use the reduced ETD2 scheme, the RK2 scheme, otherwise, as shown in Figure 2. Detailed AETD2 algorithm is given in Algorithm 2.

**Algorithm 2:** AETD2 algorithm

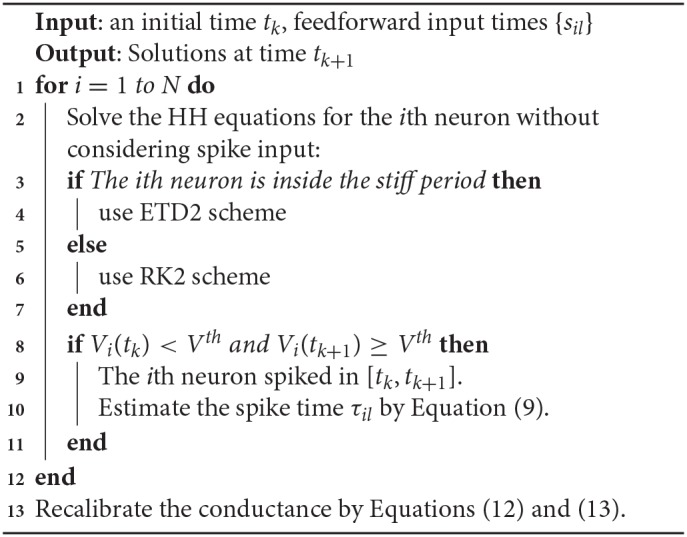

## 3. Results

We consider an all-to-all connected network of 80 excitatory and 20 inhibitory neurons driven by Poisson feedforward input. For the ease of illustration, we choose the Poisson input strength *f* = 0.06 mS·cm^−2^ and input rate ν = 300 Hz, and the coupling strength between neurons are chosen as *S* = 0.2 mS·cm^−2^ throughout this work, unless indicated otherwise. However, our algorithm can be applied to HH neural networks under a variety of dynamical regimes.

**Figure 3:**
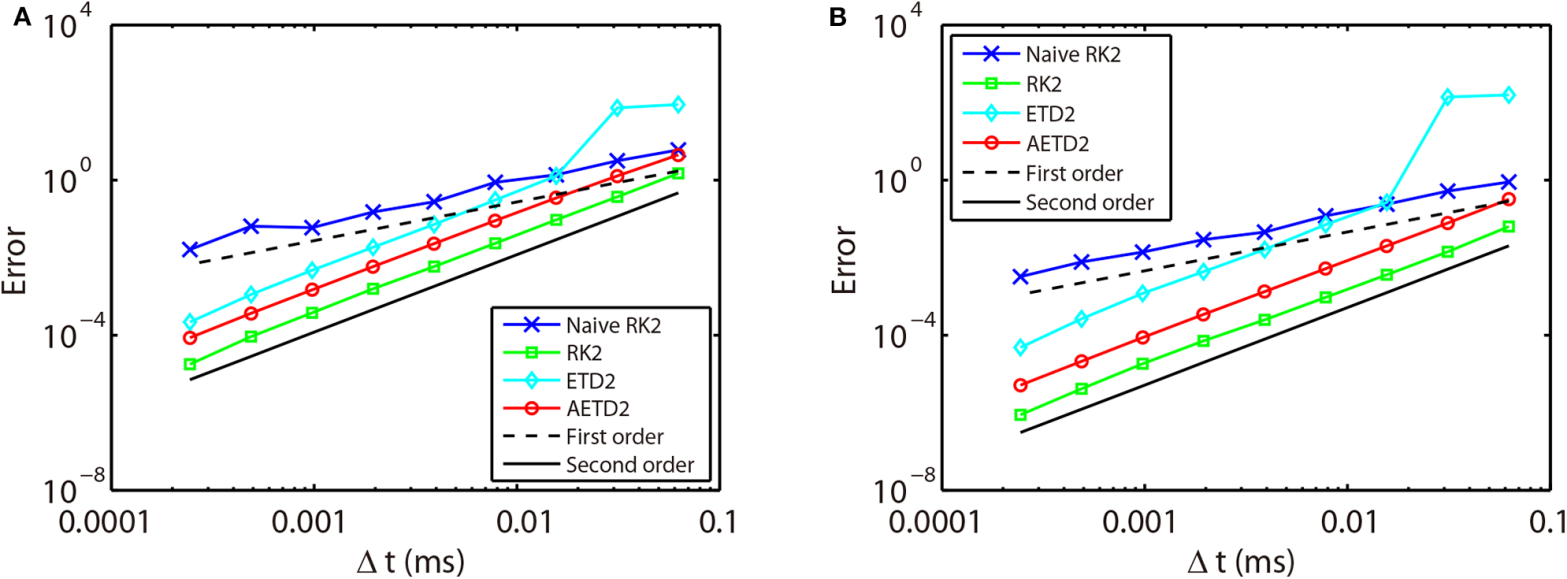
Errors of membrane potentials **(A)** and the last spike time of each neuron **(B)** in the all-to-all connected network when it is evolved using various time steps. Blue crosses are naive RK2 method without performing the linear interpolation for the estimate of the spike times. Green squares are RK2 method, cyan diamonds are ETD2 method, and red circles are AETD2 method. The last three methods all perform the linear interpolation to estimate the spike times. The dashed line and the solid line indicate the numerical convergence of the first-order and the second-order, respectively. The total run time *T* = 2, 000 ms.

First, we verify the second-order numerical accuracy by performing convergence tests. A high precision solution is obtained by using RK2 method with a sufficiently small time step Δ*t* = 1 × 10^−6^ ms and is denoted by a superscript “high.” It is compared with the solutions computed by the RK2, ETD2, and AETD2 methods with various values of larger time steps Δ*t* = 2^−4^, 2^−5^, …, 2^−12^ ms which is denoted by a superscript “Δ*t*.” Errors of membrane potentials at final run time *T* = 2, 000 ms and the last spike time of each neuron are computed:

(23) $$\begin{array}{r} {\text{Error}}_{V}=\sqrt{\underset{i}{\sum }{\left(V_{i}^{\left(\Delta t\right)}\left(T\right)-V_{i}^{\left(\text{high}\right)}\left(T\right)\right)}^{2}}, \end{array}$$

(24) $$\begin{array}{r} {\text{Error}}_{\tau }=\sqrt{\underset{i}{\sum }{\left(\tau_{il^{*}}^{\left(\Delta t\right)}-\tau_{il^{*}}^{\left(\text{high}\right)}\right)}^{2}}, \end{array}$$

where $\tau_{il^{*}}$ indicates the last spike time of the *i*th neuron during the run time interval. As shown in Figure 3, if one naively assigns the end of time step as the spike times in the RK2 method (naive RK2), the numerical accuracy of the membrane potentials and spike times can only be of the first-order. In contrast, if one determines the spike times by linear interpolation and recalibrate the conductances accordingly, all the RK2, ETD2, and AETD2 methods can achieve a second-order numerical accuracy. In addition, we find that the ETD2 method has much larger error compared with the RK2 and AETD2 methods using the same time step as shown in Figure 3. When using a time step larger than Δ*t* = 2^−6^ = 0.0156 ms, the ETD2 method performs even worse than the naive RK2 method. The underlying reason is that the HH equations are almost non-stiff in the non-spike period, but the decomposition in Equation (14) induces a relatively large stiffness for the nonlinear term as discussed previously.

**Figure 4:**
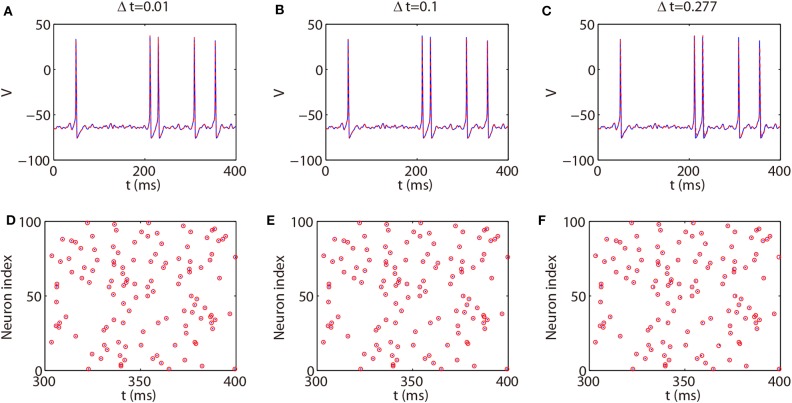
Comparing the AETD2 method with the RK2 method. (Top) Traces of membrane potential of an HH neuron in the all-to-all connected network. (Bottom): Raster plots of the network spikes. The blue solid curves and dots indicate the results by the RK2 method with time step Δ*t* = 0.01 ms, while the red dashed curves and circles indicate the results by the AETD2 method. The time steps for the AETD2 method are Δ*t* = 0.01, 0.1, 0.277 ms for **(A,D)**, **(B,E)**, and **(C,F)**, respectively.

**Figure 5:**
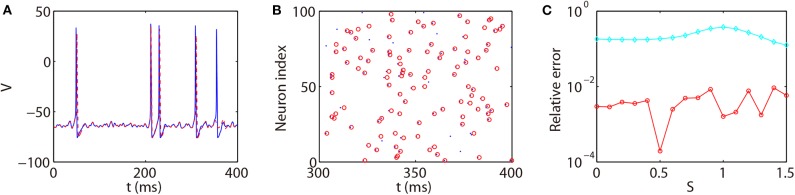
Comparing the ETD2 and AETD2 methods with the RK2 method. **(A)** Voltage trace of the same HH neuron used in Figure 4. **(B)** Raster plot of the network spikes. The blue solid curve and dots indicate the results by the RK2 method with time step Δ*t* = 0.01 ms while the red dashed curve and circles indicate the results by the ETD2 method with time step Δ*t* = 0.277 ms. The coupling strength is *S* = 0.2 mS·cm^−2^. **(C)** Relative error in the mean firing rates between the ETD2 and RK2 methods (cyan diamonds), and that between the AETD2 and RK2 methods (red circles) for different choice of the coupling strength. Both the ETD2 and AETD2 methods use time step Δ*t* = 0.277 ms. The benchmark mean firing rate is computed by the RK2 method with a very small time step Δ*t* = 1 × 10^−6^ ms.

We next discuss the numerical performance of our AETD2 method and compare it with other different numerical methods. As shown in the top panel of Figure 4, the AETD2 method with large time steps (maximum time step Δ*t* = 0.277 ms) can obtain the same high accuracy in membrane potentials as the RK2 method using a very small time step Δ*t* = 0.01 ms. The bottom panel of Figure 4 shows the raster plots (neuron index vs. its spike time) of the spike events in the network. It can be seen that the spike times are well-captured by the AETD2 method with large time steps. In contrast, as shown in Figures 5A,B, the ETD2 method is highly inaccurate in terms of voltage traces and raster plots when the time step Δ*t* = 0.277 ms is used (the maximum time step in AETD2 method). Figure 5C shows the relative error in the mean firing rate (the average number of synaptic spikes per unit time) between the ETD2 and RK2 methods, and that between the AETD2 and RK2 methods over different values of coupling strength. It can be seen that the ETD2 method can achieve only one digit of numerical accuracy while the AETD2 method can robustly achieve more than two digits of numerical accuracy when the time step Δ*t* = 0.277 ms is used in both methods. Therefore, the ETD2 method has worse numerical performance compared with the AETD2 method.

To demonstrate the efficiency of our AETD2 method, we compare the simulation time that RK2, ETD2, and AETD2 methods take for a common total run time. We simulate the all-to-all connected network by the RK2, ETD2, and AETD2 methods on a Windows platform using an Intel i7 2.6 GHz processor (the weblink of the source codes is given in section 4), and the simulation time and numerical accuracy of mean firing rate are given in Table 1. The AETD2 method can achieve over an order of magnitude of speedup compared with the RK2 method while achieving the same high accuracy in terms of the mean firing rate.

**Table 1:** Simulation of the all-to-all connected network with a total run time *T* = 10 s.

	**RK2**			**ETD2**			**AETD2**		
***Δt* (ms)**	**CPU**	**Relative error**		**CPU**	**Relative error**		**CPU**	**Relative error**	
0.005	60.56 s	0	(13.61 Hz)	60.07 s	0	(13.61 Hz)	60.82 s	0	(13.61 Hz)
0.01	30.22 s	0	(13.61 Hz)	30.05 s	0	(13.61 Hz)	30.55 s	0	(13.61 Hz)
0.02	14.99 s	0	(13.61 Hz)	15.03 s	0.074%	(13.60 Hz)	15.30 s	0	(13.61 Hz)
0.05	***	***	***	5.95 s	0.66%	(13.52 Hz)	6.16 s	0.074 %	(13.62 Hz)
0.1	***	***	***	2.94 s	2.65%	(13.25 Hz)	3.11 s	0.074 %	(13.62 Hz)
0.2	***	***	***	1.48 s	9.99%	(12.25 Hz)	1.57 s	0.15 %	(13.63 Hz)
0.277	***	***	***	1.09 s	12.56%	(11.29 Hz)	1.15 s	0.59 %	(13.69 Hz)
0.5	***	***	***	0.57 s	41.59%	(7.95 Hz)	***	***	***
1	***	***	***	0.29 s	87.07%	(1.76 Hz)	***	***	***

*The simulation time is measured in seconds. The relative error in the mean firing rate between each method using different time steps and the RK2 method using a very small time step Δt = 1 × 10^−6^ ms is measured in percentage and the mean firing rate is measured in Hz given inside the parentheses. Asterisks indicate overflow errors*.

**Figure 6:**
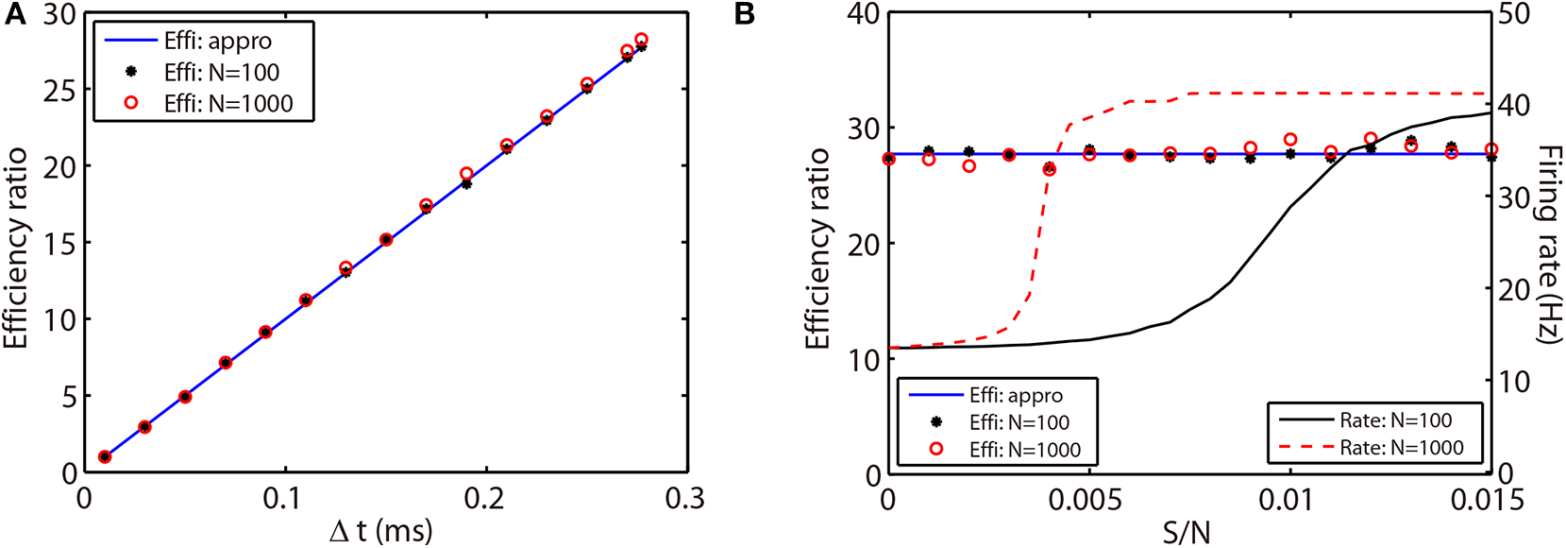
Efficiency ratio of the AETD2 method when evolving the HH neural network using various time steps **(A)** and coupling strength **(B)**. In **(A,B)**, the blue lines are efficiency ratio measured by the approximation in Equation (26), while the black stars and red circles are the efficiency ratio measured by the ratio of simulation times between the RK2 and AETD2 methods. The black stars represent the results for the all-to-all connected HH neural network of 80 excitatory and 20 inhibitory neurons, while the red circles represent the results for an HH neural network of 800 excitatory and 200 inhibitory neurons randomly connected with probability 25%. The black solid and red dashed curves in **(B)** are the mean firing rates in the smaller network of 100 neurons and larger network of 1,000 neurons, respectively. The coupling strength in **(A)** is *S*/*N* = 0.002 mS·cm^−2^ and the time step for AETD2 method in **(B)** is Δ*t* = 0.277 ms. The time step for RK2 method is 0.01 ms and total run time is *T* = 50 seconds in both **(A,B)**.

In addition, we define the efficiency ratio of the AETD2 method over the RK2 method as

(25) $$\begin{array}{r} E=\frac{T_{\text{RK2}}}{T_{\text{AETD2}}} \end{array}$$

where *T*_RK2_ and *T*_AETD2_ indicate the simulation times of the RK2 and AETD2 methods, respectively, for the HH neural network to evolve the run time *T*. Note that the RK2 and ETD2 methods take almost the same simulation time when using the same small time step as shown in Table 1. Thus, the above efficiency ratio can be approximated by the ratio of the total number of time steps each method requires as

(26) $$\begin{array}{r} E\approx \frac{T/\Delta t_{\text{RK2}}}{T/\Delta t_{\text{AETD2}}}=\frac{\Delta t_{\text{AETD2}}}{\Delta t_{\text{RK2}}}, \end{array}$$

where Δ*t*_RK2_ and Δ*t*_AETD2_ indicate the time steps used in the RK2 and AETD2 methods, respectively. To demonstrate that the above efficiency ratio is independent of the network connectivity, size, and dynamical regimes, we evolve the all-to-all connected network of 80 excitatory and 20 inhibitory neurons and a randomly connected network of 800 excitatory and 200 inhibitory neurons with a variety choice of coupling strength. Not surprisingly, the efficiency ratio approximated by Equation (26) agrees well with the one measured by the ratio of simulation time between the RK2 and AETD2 methods in both two networks as shown in Figure 6. Hence, the efficiency ratio of the AETD2 method relies on only the size of evolved time steps.

## 4. Discussion

We have presented an adaptive second-order ETD method to evolve the pulse-coupled HH neural network. Our AETD2 method can solve the stiff problem in the HH equations when an HH neuron generates an action potential (spike period). It can use a large time step to raise computational efficiency while accurately capturing dynamical properties of HH neurons such as the trace of membrane potentials, spike times of each neuron, and the mean firing rate. We point out that our AETD2 method can robustly enlarge time steps and raise computational efficiency over one order of magnitude compared with the RK2 method. This high efficiency seems to be independent of parameter choice of connectivity structure, dynamical regimes, or network size.

Our adaptive ideas of ETD methods can be applied to dynamical systems with stiff and non-stiff periods. In addition, we point out that the ETD scheme in our AETD2 algorithm can be chosen in a variety of forms according to the properties of dynamical systems. Here, we use the ETD2 scheme derived by approximating the integration in Equation (19) with RK time stepping. Other forms of numerical schemes can also be used to approximate the integration. For example, one can use a liner interpolation to approximate the nonlinear part in Equation (14) to obtain another form of ETD2 scheme. Besides, one can derive an ETD scheme following the idea proposed in Börgers and Nectow (2013) by linearly approximating the HH equations. The derived ETD scheme is proven to be unconditionally stable for HH system in Börgers and Nectow (2013). All these different ETD schemes can be easily embedded into our AETD2 method in the same way as given in Algorithm 2. For example, we can embed the ETD formula proposed in Börgers and Nectow (2013) into the AETD2 method to evolve the reduced Traub Miles (RTM) neural networks (Ermentrout and Kopell, 1998; Olufsen et al., 2003; Börgers and Nectow, 2013). The dynamical equations for an RTM neuron is almost the same as that for an HH neuron except that the gating variable *m* is described by *m*_*i*_ = α_*m*_(*V*_*i*_)/(α_*m*_(*V*_*i*_)+β_*m*_(*V*_*i*_)). The forms of α and β for the RTM neurons are set as: α_*m*_(*V*_*i*_) = 0.32(*V*_*i*_+54)/(1−exp(−(*V*_*i*_+54)/4)), β_*m*_(*V*_*i*_) = 0.28(*V*_*i*_+27)/(exp((*V*_*i*_+27)/5)−1), α_*h*_(*V*_*i*_) = 0.128exp(−(*V*_*i*_+50)/18), β_*h*_(*V*_*i*_) = 4/(1+exp(−(*V*_*i*_+27)/5)), α_*n*_(*V*_*i*_) = 0.032(*V*_*i*_+52)/(1−exp(−(*V*_*i*_+52)/5)), and β_*n*_(*V*_*i*_) = 0.5exp(−(*V*_*i*_+57)/40).

**Figure 7:**
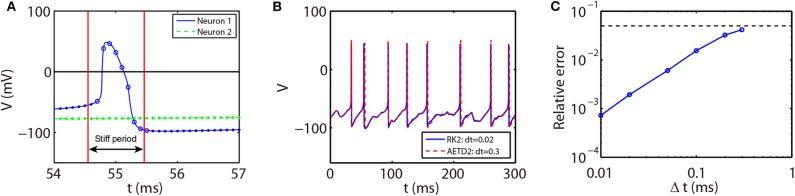
The AETD2 method for the RTM neural network. **(A)** Illustration of the AETD2 method. We take a relatively high firing threshold *V*^th^ = 0 mV for the RTM neurons indicated by the solid horizontal line. The stiff period is defined as the region where |*dV*/*dt*|≥20. The circles and dots indicate the time nodes where we use the ETD2 and RK2 schemes, respectively. **(B)** Traces of membrane potential of an RTM neuron. The blue solid and red dashed curves indicate the results by the RK2 method with time step Δ*t* = 0.02 ms and the AETD2 method with time step Δ*t* = 0.3 ms, respectively. **(C)** Relative error in the mean firing rates between the AETD2 and RK2 methods for different choice of time steps used in the AETD2 method for an all-to-all connected RTM network of 80 excitatory and 20 inhibitory neurons with Poisson input. The dashed horizontal line indicates 5% error, relative error = 0.05. The benchmark mean firing rate is computed by the RK2 method with a very small time step Δ*t* = 1 × 10^−6^. The parameters for RTM model is *C* = 1μF·cm^−2^, *V*_*Na*_ = 50 mV, *V*_*K*_ = −100 mV, *V*_*L*_ = −67 mV, *G*_*Na*_ = 100 mS·cm^−2^, *G*_*K*_ = 80 mS·cm^−2^, and *G*_*L*_ = 0.1 mS·cm^−2^.

Note that the rising phase of action potentials for the RTM neurons is extremely short, around 0.03 ms as shown in Figure 7A. In such a situation, it may not be appropriate to choose the spike time (when the voltage reaches the firing threshold) as the starting point of the stiff period in the AETD2 method as shown in Figure 7A since large numerical error will be introduced, especially when a large time step is used, e.g., time step Δ*t* = 0.3 ms. This is because the system is evolved by the RK2 scheme during the time step that contains the rapid rising region of the neuron's action potential. Therefore, to achieve high numerical accuracy, the interval of the stiff period should cover the rapid rising region. To achieve this, we then define the stiff period of the RTM neurons as the region where the magnitude of the slope of voltage is over a proper threshold as shown in Figure 7A. We point out that our AETD2 method using a large time step can still accurately capture the membrane potential traces and the mean firing rates compared with the RK2 method using a small time step as shown in Figures 7B,C. Therefore, the definition of the stiff period in our AETD2 method can be flexibly determined based on dynamical properties of studied systems.

In this work, the numerical accuracy of our AETD2 method is second-order. In some situations, high accurate traces of membrane potentials may be required, especially the accurate shapes of action potentials (Traub et al., 2001; Kopell and Ermentrout, 2004). Therefore, one future work may be the design of the fourth-order ETD method. As illustrated above, due to the discontinuity arising from the pulse-coupled dynamics, an even more careful recalibration needs to be designed to achieve fourth-order numerical accuracy.

Finally, we point out that our AETD2 method can be easily extended to networks of other HH type neurons (Pospischil et al., 2008). And our AETD2 method can also robustly achieve high numerical accuracy and efficiency. In addition, our method is naturally a parallel algorithm which can be applied to simulations of large-scale neural network dynamics. For reproducibility of our results by other researchers, all the source codes written in C++ can be accessed at http://github.com/KyleZhongqi/ETD2_HH.

## Data Availability Statement

The datasets generated for this study are available on request to the corresponding author.

## Author Contributions

ZT and DZ designed the research, wrote and code, ran the simulations, analyzed the results, and wrote the paper.

## Conflict of Interest

The authors declare that the research was conducted in the absence of any commercial or financial relationships that could be construed as a potential conflict of interest.
